# *Helicobacter pylori* in Human Stomach: The Inconsistencies in Clinical Outcomes and the Probable Causes

**DOI:** 10.3389/fmicb.2021.713955

**Published:** 2021-08-17

**Authors:** Sneha Mary Alexander, Radhakrishnan Jayalakshmi Retnakumar, Deepak Chouhan, Thillai Natarajan Barani Devi, Sanjai Dharmaseelan, Krishnadas Devadas, Namrata Thapa, Jyoti Prakash Tamang, Sangey Chhophel Lamtha, Santanu Chattopadhyay

**Affiliations:** ^1^Rajiv Gandhi Centre for Biotechnology, Trivandrum, India; ^2^Centre for Doctoral Studies, Manipal Academy of Higher Education, Manipal, India; ^3^Department of Gastroenterology, Government Medical College, Trivandrum, India; ^4^Biotech Hub, Department of Zoology, Nar Bahadur Bhandari Degree College, Gangtok, India; ^5^Department of Microbiology, Sikkim University, Gangtok, India; ^6^STNM Hospital, Gangtok, India

**Keywords:** gastric cancer and peptic ulcer, *Helicobacter pylori*, other factors, clinical outcomes, inconsistancy

## Abstract

Pathogenic potentials of the gastric pathogen, *Helicobacter pylori*, have been proposed, evaluated, and confirmed by many laboratories for nearly 4 decades since its serendipitous discovery in 1983 by Barry James Marshall and John Robin Warren. *Helicobacter pylori* is the first bacterium to be categorized as a definite carcinogen by the International Agency for Research on Cancer (IARC) of the World Health Organization (WHO). Half of the world’s population carries *H. pylori*, which may be responsible for severe gastric diseases like peptic ulcer and gastric cancer. These two gastric diseases take more than a million lives every year. However, the role of *H. pylori* as sole pathogen in gastric diseases is heavily debated and remained controversial. It is still not convincingly understood, why most (80–90%) *H. pylori* infected individuals remain asymptomatic, while some (10–20%) develop such severe gastric diseases. Moreover, several reports indicated that colonization of *H. pylori* has positive and negative associations with several other gastrointestinal (GI) and non-GI diseases. In this review, we have discussed the state of the art knowledge on “*H. pylori* factors” and several “other factors,” which have been claimed to have links with severe gastric and duodenal diseases. We conclude that *H. pylori* infection alone does not satisfy the “necessary and sufficient” condition for developing aggressive clinical outcomes. Rather, the cumulative effect of a number of factors like the virulence proteins of *H. pylori*, local geography and climate, genetic background and immunity of the host, gastric and intestinal microbiota, and dietary habit and history of medicine usage together determine whether the *H. pylori* infected person will remain asymptomatic or will develop one of the severe gastric diseases.

## Introduction

**Figure 1:**
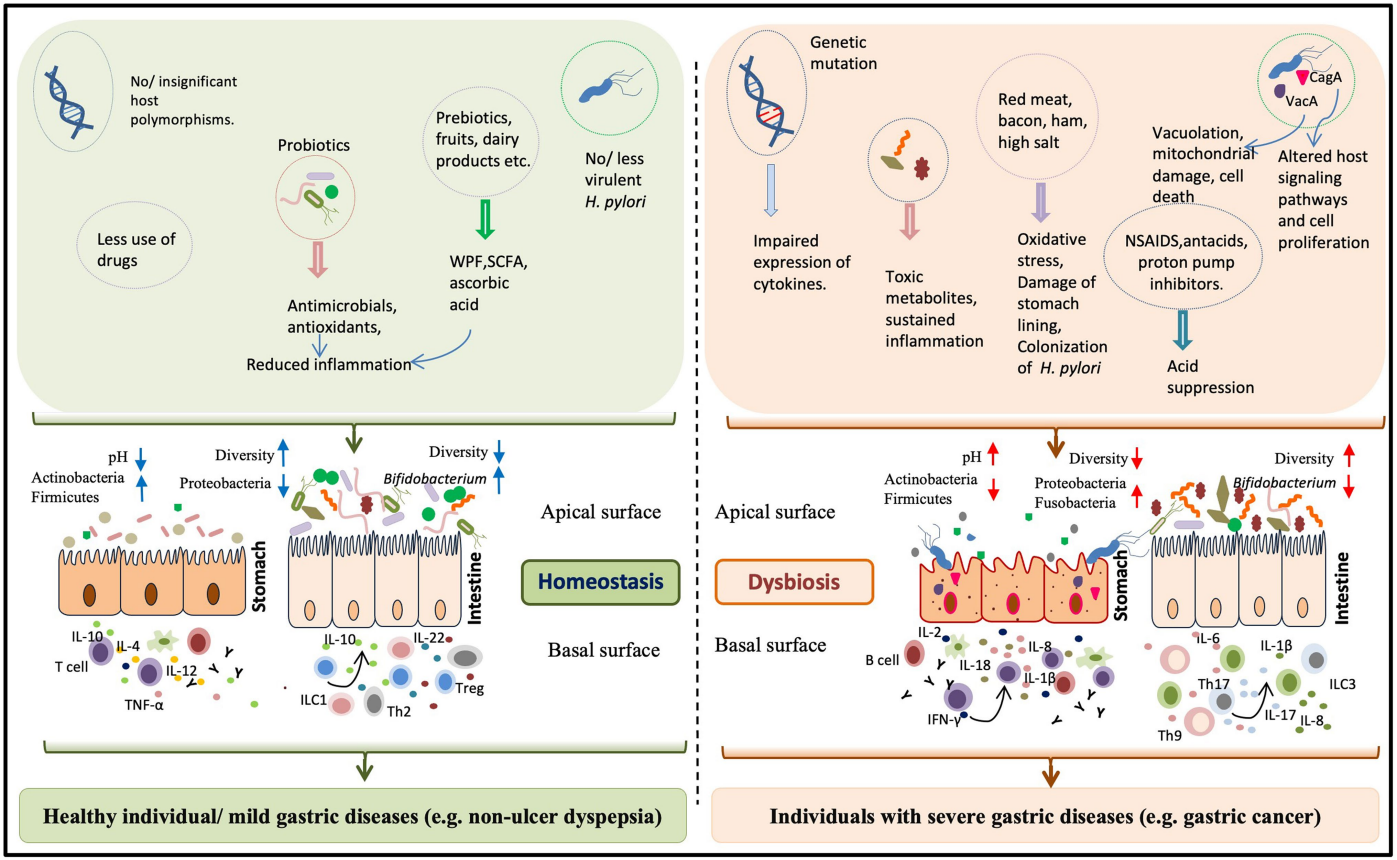
The impact of “*H. pylori* factors” and “other factors” on the gastric health and diseases. The gastric health is determined by the combination of microbial, host, and environmental factors. Normally gastric inflammation remains under control by the constant interaction between the inhabiting microbes and the host immune system. The beneficial microbes in the form of probiotics (producing antimicrobials and anti-oxidants) or in ingested ethnic fermented foods and the prebiotics play major roles in maintaining the homeostasis within the gastric milieu. For a healthy stomach, where the pH is low, the colonization is restricted to microbial phyla like Actinobacteria and Firmicutes. But, the long term colonization of *H. pylori*, apart from creating gastric damages due to its virulence factors like vacuolating cytotoxin A (VacA) and CagA, also leads to the loss ofgastric parietal cells and the gastric pH increases. In the altered gastric pH, several harmful bacteria (e.g., several members of the phylum Proteobacteria) become predominant. Several other factors have the potentials to disturb the gastric homeostasis. For instance, impaired expression of host immune regulators, increased consumption of red meat, and excessive use of non-steroidal anti-inflammatory drug (NSAID) and acid suppressors can enhance the pro-inflammatory response. Dysbiosis of the intestinal microbiota, particularly lower abundance of *Bifidobacterium* species has recently been linked to the *H. pylori* associated severe gastric diseases.

Two gastric diseases, peptic ulcer (246,700 deaths/year) and gastric cancer (782,685 deaths/year), together are responsible for over a million of global deaths annually (Moraga et al., 2017; Bray et al., 2018). Both diseases are associated with the colonization of *Helicobacter pylori* in stomach (Table 1). *Helicobacter pylori* infection is also associated with a few relatively milder gastrointestinal (GI) diseases like non-ulcer dyspepsia (NUD) and gastritis, which often remains unnoticed (Table 1). Even several non-GI diseases like myocardial infarction, diabetes mellitus, and iron deficiency anemia are reported to be linked to *H. pylori* infection (Table 1; Ortiz et al., 2014). On the other hand, it has also been proposed that *H. pylori* infection is negatively associated with several upper-GI diseases like gastroesophageal reflux disease (GERD), Barrett’s esophagus, and esophageal cancer and also with some non-GI diseases like asthma (Table 1; Fallone et al., 2000; Wang et al., 2013; Rubenstein et al., 2014). Till date, it is not understood how *H. pylori* infection is positively and negatively associated with so many GI and non-GI diseases, but the *H. pylori* encoded virulence factors and their mechanisms of actions in the context of peptic ulcer and gastric cancer are well studied (Yamaoka, 2010; Malfertheiner et al., 2014). However, even for the gastroduodenal diseases, the *H. pylori* infection and the virulence factors of *H. pylori* fail to explain the inconsistencies in clinical outcomes that are observed within the *H. pylori* infected population. For example, nearly 4.4 billion people, which means more than half of the world’s population is infected with *H. pylori* and the majority of the *H. pylori* strains carry the virulence genes, but only a small fraction, usually 10–20%, develop the severe gastric diseases like peptic ulcer disease and gastric cancer (Dorer et al., 2009; Hooi et al., 2017). It is not known why 80–90% of the *H. pylori* infected population remains asymptomatic. Nonetheless, the observed discrepancies in *H. pylori* infections and the gastric diseases clearly suggest definite involvements of some “other factors” in determining the clinical status of the host. In this review, we discussed the state of the art knowledge regarding the “*H. pylori* virulence factors” and the “other factors” like host genetics, host immunity, geography, climate, GI microbiota, diet and medications, which together contribute to either the “development and progression” or the “prevention” of peptic ulcer and gastric cancer (Figure 1).

**Table 1:** Correlation between *H. pylori* colonization and various disease conditions.

Diseases		Association with *H. pylori*	Reference
Gastrointestinal (GI) diseases	Gastric MALT lymphoma	+ve	Watari et al., 2014
	Gastritis	+ve	Watari et al., 2014
	Duodenal ulcer	+ve	Narayanan et al., 2018
	Gastric ulcer	+ve	Narayanan et al., 2018
	Gastric adenocarcinoma	+ve	Correa and Piazuelo, 2011
	Esophageal cancer	-ve	Polyzos et al., 2018
	Gastro-Esophageal Reflux Disease (GERD)	-ve	Labenz et al., 1997; Fallone et al., 2000
	Barrett’s esophagus	-ve	Rubenstein et al., 2014
Non- gastrointestinal (non-GI) diseases	Iron deficiency anemia	+ve	Ortiz et al., 2014; Sato et al., 2015
	Diabetes mellitus	+ve	Yang et al., 2014; Chen et al., 2015
	Coronary artery diseases	+ve	Yu et al., 2017
	Idiopathic thrombocytopenic purpura	+ve	Franchini and Veneri, 2004; Kikuchi et al., 2011
	Allergy	-ve	Lee et al., 2015
	Asthma	-ve	Zevit et al., 2012; Wang et al., 2013
	Multiple sclerosis	-ve	Li et al., 2007b; Cook et al., 2015
	Coeliac disease	-ve	Diamanti et al., 1999; Ciacci et al., 2000
	Inflammatory bowel diseases (IBD)	-ve	Robinson, 2015

## *Helicobacter pylori* Virulence

Since *H. pylori* is sensitive to low pH, its survival in highly acidic gastric milieu is challenging. However, the bacterium overcomes this challenge by producing a 550 kD a multimeric nickel-containing enzyme, urease, which catalyzes the hydrolysis of urea into carbonic acid and ammonia (Table 2). The released ammonia increases the pH and provides a protective local environment near *H. pylori*. Simultaneously, the urease can also activate monocytes and polymorphonuclear leucocytes leading to inflammatory response and epithelial damage (Mobley, 1996).

**Table 2:** Virulence factors of *H. pylori* and their polymorphisms associated with gastric diseases.

Virulence factor	Function	Key polymorphisms/alleles/variants	Association with gastric diseases	Reference
Urease	Neutralizes gastric acidity by converting urea to basic ammonia. Induce inflammatory damage to gastric epithelium by activating various immune cells	-	Essential for bacterial colonization and further stages of *H. pylori* pathogenesis.	Mobley, 1996
BabA	Facilitates adhesion of *H. pylori* to gastric epithelial cells.	BabA-L: No Le^b^ binding activity	Associated with high gastric mucosal damage and gastric cancer	Fujimoto et al., 2007
		BabA-H: Show Le^b^ binding activity	Associated with mild gastric mucosal damage	
		BabA-ve: No Le^b^ binding activity		
CagA	Virulence protein which alters host cell signaling pathways	ESS-CagA (EPIYA-D) Binds more strongly with SHP2 than WSS type and potentially activates the downstream pathways	Positively associated with aggressive gastric diseases.	Higashi et al., 2002
		K636N mutation	Higher risk of severe pathology	Ulloa-Guerrero et al., 2018
CagL	Type IV secretion system (TFSS) protein that binds with integrinβ1 receptor on host cell.	Y58/E59 polymorphism	Increased risk of gastric cancer.	Yeh et al., 2019
VacA	Pore forming toxin that induces vacuolation, mitochondrial damage and cell death.	*vacA s1m*	High risk of gastric precancerous lesions	Chang et al., 2018
		*vacA c1and d1*	Increased risk of gastric cancer	Thi Huyen Trang et al., 2016
DupA	Duodenal ulcer promoting protein	-	Increased risk of duodenal ulcer.	Lu et al., 2005
OipA	Enhance IL-8 secretion and induces inflammation	Functional *oipA* “on” status	Increased risk of peptic ulcer and gastric cancer	Liu et al., 2013
HPne4160	Non-coding RNA that regulate expression of outer membrane protein (OMP) and CagA	T-repeats present upstream of HPnc4160	Silences HPnc4160 resulting in increased expression of OMP and CagA	Kinoshita-Daitoku et al., 2021

The next critical event for successful colonization of *H. pylori* is to establish a firm adhesion with the gastric epithelial cells, which is mediated by a group of outer membrane adhesins that interact with molecules on the host cell surface. Blood group antigen binding adhesin A (BabA) and Blood group antigen binding adhesin B (BabB) are well-characterized bacterial adhesins that interact with the host Lewis B blood group antigens (Le^b^; Boren et al., 1993). *Helicobacter pylori* strains can be classified as BabA high producers (BabA-H) with Le^b^ binding activity, BabA low producers (BabA-L) without Le^b^ binding activity, and BabA-negative strain (*babA2*-genonegative; Table 2; Fujimoto et al., 2007). Interestingly, however, infections with BabA-L strains are associated with the highest mucosal injury and have the highest risk of gastric cancer (Fujimoto et al., 2007; Chang et al., 2018). In populations where A, B and O blood groups are abundant, majority of the isolated *H. pylori* can bind to fucosylated blood group antigens A, B, and O (generalist). But, in the South American Amerindian population, where the O blood group is predominant, 60% of *H. pylori* strains binds best to the O antigen (specialist), showing that the adherence pattern is influenced by the regional abundance of A, B, and O blood groups (Aspholm-Hurtig et al., 2004). This adaptive binding is achieved by a single amino acid substitution in the Diversification Loop1(DL1) in BabA (Moonens et al., 2016). The Le^b^-BabA interaction is inhibited at low pH and is restored with acid neutralization. This acid responsiveness, which depends on the pH sensor sequence in BabA, differs among strains suggesting that *H. pylori* strains may get adapted to the distinct acid secretion pattern of each individual (Bugaytsova et al., 2017). The BabA also potentiates the activity of the *H. pylori* Type IV secretion system (TFSS; Ishijima et al., 2011). The *H. pylori* outer inflammatory protein A [OipA, also known as *Helicobacter* outer membrane protein (OMP) H or HopH] is an OMP that is involved in adhesion and promotes IL-8 secretion. Strains with *oipA* “on” status are associated with increased colonization density, neutrophil infiltration and IL-8 production. In addition, significant association has been found between the *oipA* “on” status and the risk of peptic ulcer and gastric cancer (Table 2; Liu et al., 2013). Other than OipA, *H. pylori* also possesses other OMPs like sialic acid binding adhesin (SabA, also known as *Helicobacter* OMP P/HopP), *Helicobacter* OMP Q (HopQ), *Helicobacter* OMP Z (HopZ), and *H. pylori* outer membrane (Hom) family proteins, such as HomA, HomB, HomC, and HomD. The expression of SabA correlates inversely with gastric pH and helps *H. pylori* adapt with the changing gastric environment. It was reported that HopQ has essential role in the translocation of CagA into host cell. Among the Hom proteins, HomB is capable of inducing IL-8 secretion (Xu et al., 2020). Adherence-associated lipoprotein A and B (AlpA/AlpB) aid *H. pylori* to bind with gastric epithelial cell and facilitate colonization and biofilm formation. It also induces expression of inflammatory mediators like IL-6 and IL-8. A poorly studied adhesin, LacdiNAc-specific adhesin (LabA) also mediates *H. pylori* adhesion to gastric epithelial cells (Baj et al., 2021). Chronic colonization of *H. pylori*, as evident from DNA fingerprinting studies of strains isolated from sequential biopsies, is critical in the progression of severe gastric diseases (Taylor et al., 1995). Recent study has shown that adaptability of *H. pylori* to the gastric environment is regulated by a small non-coding RNA HPne4160. Decreased expression of HPne4160 during chronic infection results in increased expression of OMPs and CagA (Kinoshita-Daitoku et al., 2021).

The genes that encode the TFSS and the virulence protein CagA are located within the ~40 kb *cag*-pathogenicity island (*cag*PAI). The CagA N-terminal domain also contains a binding site for the α5β1 integrin and this interaction is considered to be a prerequisite for the delivery of CagA into the gastric epithelial cells (Kaplan-Türköz et al., 2012). Once inside the cell, the phosphatidyl serine (PS) interacts with the Lys-Xn-Arg-X-Arg (K-Xn-R-X-R) motif in the N-terminal domain of CagA and anchors CagA to the inner surface of cell membrane (Takahashi-Kanemitsu et al., 2020). In this motif, the Lys and Arg (R619 and R621) residues are highly conserved and exposed for both Western (e.g., 26,695 and G27, J99) and East Asian (e.g., F75) strains. Conversely, the position 636 is highly variable and the K636N mutation is associated with severe pathology (Table 2; Ulloa-Guerrero et al., 2018). Inside the cell, the CagA gets phosphorylated and forms complexes with the Src-homology 2 (SH2) domains present in the proteins like SHP2, Grb2, and CSK and alters a number of host signaling pathways that leads to abnormal cytoskeletal changes, cell proliferation, and differentiation and induces the secretion of pro-inflammatory cytokines like IL-8 through NF-κB pathway (Papadakos et al., 2013; Hatakeyama, 2014). The key phosphorylation, which leads to these cascades of downstream events, occurs at the C-terminal domain of CagA. This domain contains a conserved phosphorylayable tyrosine residue within the motifs composed of five amino acids, Glu-Pro-Ile-Tyr-Ala (EPIYA). Based on the variation in the flanking regions and the orders of spacers, the CagA-EPIYA motifs can be classified into A, B, and either C or D types. The first EPIYA motif is called as EPIYA-A, which is followed by the EPIYA-B and then either EPIYA-C or EPIYA-D. The C segment (usually 1–3 copies) is the characteristic of Western CagA (WSS), while the D segment (usually one copy) is seen in East-Asian CagA (ESS; Higashi et al., 2002). For the WSS-CagA (EPIYA-C), the degree of phosphorylation is proportional to the copy number of EPIYA-C, which influences the strength of activation of downstream pathways. However, the ESS-CagA (EPIYA-D) has stronger affinity for SHP2, which potentiates the downstream effector pathways leading to aggressive diseases (Table 2). In addition, the level of expression of CagA is found to vary among strains. Mutations in other genes in the *cagPAI* can also affect the virulence. For example, *H. pylori* strains with Y58/E59 polymorphism in the CagL carry higher risk for inducing a gastric cancer (Table 2; Yeh et al., 2019).

The vacuolating cytotoxin A (VacA) is a pore forming secreted toxin, which is composed of N-terminal (p33) and C-terminal (p55) fragments, binds to the receptors like receptor protein tyrosine phosphatase beta (RPTP-β; Yahiro et al., 2003), epidermal growth factor receptor (EGFR), and sphingomyelin and gets internalized through a clathrin independent endocytosis mechanism (Gauthier et al., 2005). The internalized VacA produces large acidic vacuoles inside the cells (Utsch and Haas, 2016). In addition, the p33 subunit of VacA enters the mitochondria and induces cytochrome C release *via* Bax activation and promotes cell death (Yamasaki et al., 2006). The *vacA* gene encodes a precursor protein with a “signal sequence” (*s*), a “mid-region” (*m*), and a “C-terminal region.” Much later, another region between the *s* and *m* regions have been identified and named as intermediate (*i*) region (Table 2). The *vacA* gene is highly polymorphic and each region (*s*, *i*, *m*, *c*, and *d*) has at least two alleles; for *vacAs*, *s1* (subdivided into *s1a* and *s1b*) and *s2*; for *vacAi*, *i1* (subdivided into *i1a* and *i1b*) and *i2* and for *vacAm*, *m1* (subdivided into *m1a*, *m1b*, and *m1c*) and *m2*. The *vacAs1i1m1* allelic combination is strongly linked with the presence of *cagA*, resulting in a highly virulent strain type (*vacAs1i1m1cagA*+; Sugimoto et al., 2009). Infection with strains that express both *cagA* and *vacAs1m1* simultaneously has 4.8-fold more risk of forming gastric precancerous lesions than the individuals infected with benign *H. pylori* strains (e.g., *vacAs2m2cagA*−; Table 2; Chang et al., 2018). Apart from the diversity regions “*s*,” “*m*” and “*i*,” *vacA* possess “*c*” and “*d*” regions, but their role in the pathogenesis is still poorly understood. The *c1* and *d1* genotypes can be used as biomarkers as they are linked with the increased risk of gastric cancer. Also, the *d1/c1* strains are associated with secreted vacuolating cytotoxin type encoding *s1m1i1* and *d2/c2* strains are linked with non-secreted vacuolating cytotoxin type encoding *s2/m2/i2* genotypes (Thi Huyen Trang et al., 2016).

Studies from Asian countries showed that the presence of duodenal ulcer promoting gene A (*dupA*) is associated with increased risk of duodenal ulcer with heavy neutrophil infiltration and increased IL-8 expression in the antrum (Table 2; Yamaoka et al., 1998, 1999; Lu et al., 2005). Conversely, its presence has negative correlation with the risk of gastric atrophy, intestinal metaplasia, and gastric cancer (Hussein, 2010; Shiota et al., 2010). Similarly neutrophil activating protein (NAP) of *H. pylori* stimulates the infiltration of neutrophil to the gastric epithelium and leads to the production of reactive oxygen species (ROS; Baj et al., 2021).

The persistence and long term colonization of *H. pylori* in the gastric mucosa are facilitated by the formation of biofilm especially under stress. In order to survive the harsh environment, *H. pylori* adopts mechanisms including morphological transformation, membrane vesicles secretion, matrix production, efflux pump activity, and intermicrobial communication, leading to the formation of biofilms (Krzyżek et al., 2020). It also promotes the exchange of genetic material between subpopulations and enhances the recombination frequency (Hathroubi et al., 2018). A recent study suggests that a laboratory strain, *H. pylori* strain G27, is capable of forming biofilms on both plastic (abiotic) and gastric epithelial cells (biotic). It is also found that biofilm formation is associated with the enhanced expression of multiple genes associated with flagella formation, hydrogenase activity, and acetone metabolism (Hathroubi et al., 2020). Various studies report that the biofilm formation can enhance antibiotic resistance (Fauzia et al., 2020) especially to clarithromycin (Hathroubi et al., 2018). Further, transformation from active spiral form to coccoid form under unfavorable conditions is observed in *H. pylori*. Persistence of non-replicable, non-culturable, less active coccoid form can cause serious damage to the gastric mucosa and is usually resistant to antibiotics (Krzyżek and Grande, 2020) and is a major challenge in *H. pylori* eradication, (Ierardi et al., 2020).

Although, a number of virulence factors of *H. pylori* were identified and their potential to contribute to the formation of gastric diseases have been proved by *in vitro* and *in vivo* methods, colonization by *H. pylori* strains carrying virulence genes does not ensure aggressive clinical outcomes, particularly in some geographical regions (Chattopadhyay et al., 2002; Malfertheiner et al., 2014).

## Geography and Distinctiveness of Different *H. Pylori* Strains

The prevalence of *H. pylori* infection and associated gastric diseases vary widely with geography. In general, the prevalence of *H. pylori* infection is higher in developing countries and in resource poor settings than the developed countries. For example, the prevalence of *H. pylori* infection in Africa (79.1%), Asia (54.7%), and Latin America and Caribbean region (63.4%) are remarkably higher as compared to the prevalence in North America (37.1%) and Oceania (24.4%; Hooi et al., 2017). Interestingly, however, the indigenous populations of the developed countries like United States and Australia have relatively higher prevalence of *H. pylori* infection (Hooi et al., 2017). The geographic disparity of the prevalence of *H. pylori* infection could be due to the sanitation, urban vs. rural lifestyle, socioeconomic status, variability of the *H. pylori* strains, and antibiotic usage in the community. More intriguingly, the prevalence of gastric cancer, gastric ulcer, and duodenal ulcer do not always correlated to the prevalence of *H. pylori* infection. For instance, although the prevalence of *H. pylori* infection (80%) is very high in Africa, the incidence of gastric cancer Africa is lower and is estimated to be 4/100,000 population (Asombang et al., 2014; Smith et al., 2019). This phenomenon is known as the “African enigma” (Agha and Graham, 2005). In contrast, while the seroprevalence of *H. pylori* among Chinese and Japanese adults are 44 and 55%, respectively, the annual incidence of gastric cancers are really high (32–59 per 100,000 populations in China and 80–115 per 100,000 populations in Japan; Singh and Ghoshal, 2006).

The evidence of *H. pylori* infection has been detected in a 5,300 years old iceman mummy suggesting that *H. pylori* colonized human stomach since ancient time (Maixner et al., 2016). The first description of gastric ulcer was found as early as 4th century BCE as carvings on a pillar of the temple of Aesculapius at Epidaurus (Graham, 2014). Importantly, *H. pylori* causes long-term colonization in the human stomach, which allows the bacterium to get adapted to the particular host. Therefore, since ancient time, the bacterium has been coevolving with different human populations inhabiting different geographical regions. The present *H. pylori* strains isolated from distinct human populations in different geographical areas are distinct. At present, the *H. pylori* strains are grouped into seven distinct types: hpAfrica1, hpAfrica2, hpNEAfrica, hpEast Asia, hpAsia, hpEurope, and hpSahul (Linz et al., 2007; Lamichhane et al., 2019). The hpEurope is considered as an ancient hybrid of hpNEAfrica and hpAsia, while the hspAmerind is possibly a subpopulation of the hspEAsia (Thorell et al., 2017). However, the hspAmerind strains are now rare even within the groups having substantial Native American ancestry possibly due to the constant evolutionary pressure imposed by other competing strains (Thorell et al., 2017).

**Figure 2:**
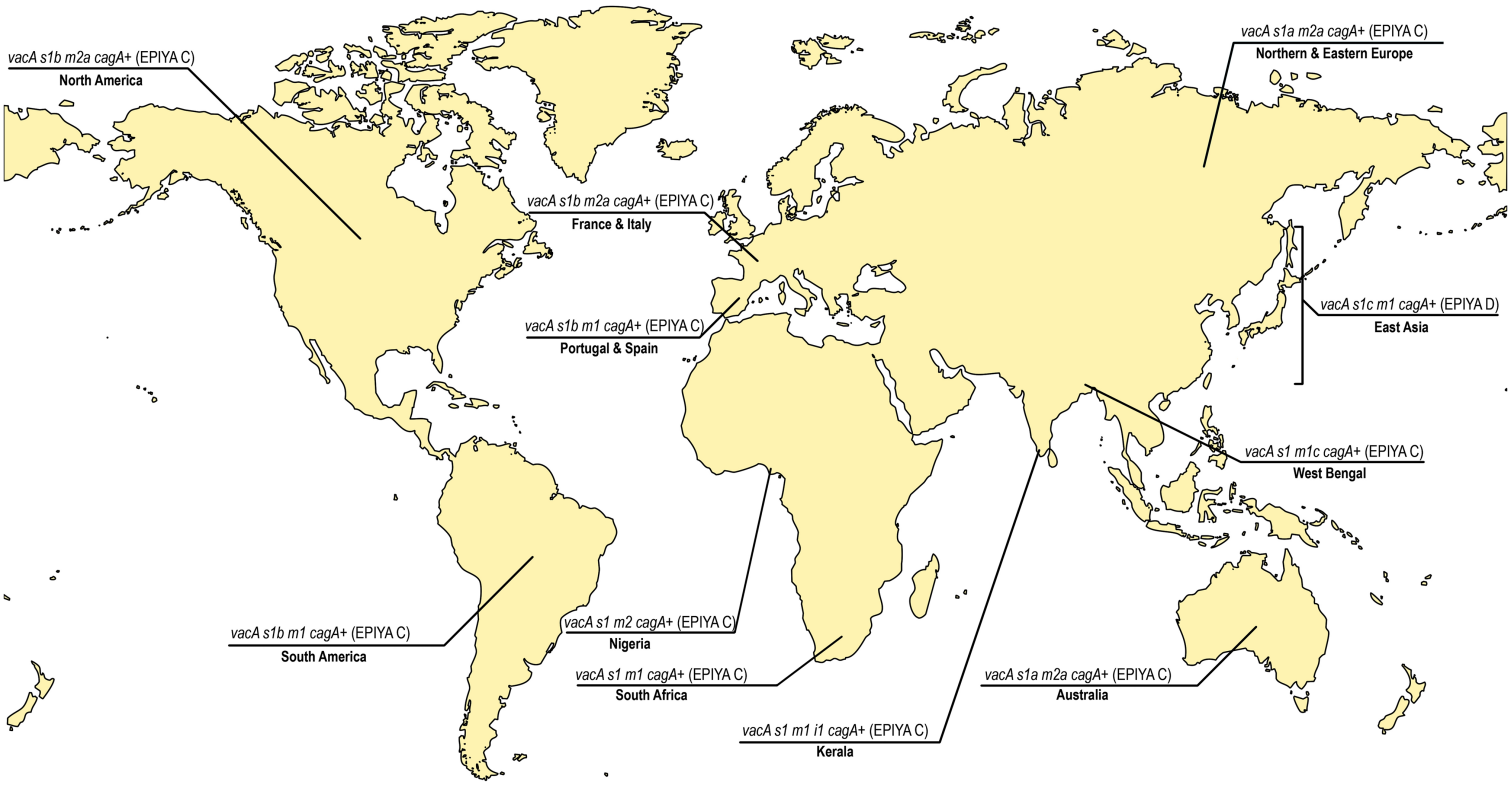
The predominant *vacA* genotypes and *cagA* status in different regions of the world. East Asia, North America, South America, Europe (France, Italy, Portugal Spain, Northern, and Eastern Europe), Africa (Nigeria and South Africa), India (West Bengal and Kerala), and Australia (van Doorn et al., 1999a; Mukhopadhyay et al., 2000; Chattopadhyay et al., 2002; Idowu et al., 2019; Ofori et al., 2019; Devi et al., 2021). The predominant *vacA* genotypes vary between and within the continents. The more virulent Glu-Pro-Ile-Tyr-Ala (EPIYA)-ABD type CagA is confined to strains circulating in East Asian countries where as the EPIYA-ABC type CagA is found in rest of the countries. The age standardized incidence of gastric cancer per 100,000 individuals in different countries has previously been reported [Eastern Asia and Eastern Europe ≥11.1; Portugal 7.3 to11.1; Spain 5 to 7.3; France 3.8 to 5; Italy 5 to 7.3; South America 7.3 to 11.1; North America 3.8 to 5; India (except West Bengal: 5 to 7.3), Australia, South Africa: 3.8 to 5;and Nigeria: <3.8; Smyth et al., 2020].

Taken together, the lineages of *H. pylori* strains are not uniformly dispersed in the world (van Doorn et al., 1999b). Like the variations in the *H. pylori* housekeeping genes, the virulence genes also vary significantly with geography. Earlier, it was noticed that the *cagA1* variant is found exclusively in strains from Europe, United States, and Australia, whereas another variant, the *cagA2*, is found in East Asian countries (van der Ende et al., 1998). It has been found that the hpAfrica2 type strains tend to lack *cag*PAI, while the 87.5% of strains from China is reported as *cagA* positive with higher frequency of *vacAi1* (85.2%) and *vacAm1* (53.6%; Duncan et al., 2013; Burucoa and Axon, 2017). Moreover, the East-Asian *H. pylori* strains carry the “D”-type segment in the C-terminal region of the CagA protein, which facilitates stronger CagA-SHP2 interactions and enhanced downstream signaling leading to aggressive gastric diseases (Higashi et al., 2002; Figure 2). This can partially explain why the prevalence of gastric cancer is highest in East-Asia and lowest in Africa. However, even in East-Asian countries, all individuals who are infected with the virulent *H. pylori* strains do not develop gastric cancer. Therefore, apart from the variations in the *H. pylori* strains in different geographical regions, additional factors like diversities in food habits, climates, and human genetic polymorphisms may also play a significant role in determining clinical outcomes.

## Climate

The different climatic and weather conditions in various parts of the world may also serve as one of the determining factors for the development of gastric diseases particularly in the context of peptic ulcer associated bleeding. *Helicobacter pylori* infection has been shown to be positively associated with daily average sunshine time and the expression of Vitamin D receptor increases in the gastric mucosa upon *H. pylori* infection (Guo et al., 2014; Lu et al., 2018). However, higher average annual temperature is negatively associated with *H. pylori* infection and the incidence of peptic ulcer in cold climate is significantly higher than in hot climates (Yuan et al., 2015; Lu et al., 2018). This might be due to the higher gastric acid secretions in response to the cold exposures (Yang et al., 2020). Experiments on the organotypic stomach slice cultures of mice showed that the cold-stress has the potential to induce gastrin expression and enhance the gastric acid secretion (Yang et al., 2020). Furthermore, the diversity of food habits in hot and in cold climatic regions may also contribute to the observed variations in the prevalence of *H. pylori* infection and the prevalence of gastric diseases (discussed later under the heading, Diet).

## Host Immune System, Genetics, and Associated Polymorphisms

The persistence of *H. pylori* in the human stomach for years is achieved by maintaining a sustained host–microbe interaction, which begins as soon as the bacteria get attached to the gastric epithelial cells. Since the genetic makeup of each individual is different, their responses to invading pathogens are also expected to vary. The initial interaction between the bacteria and innate host immune system is mediated through the pattern recognition receptors (PRRs) expressed on the gastric epithelial cells. Various toll-like receptors (TLR) are one of the key PRRs, which trigger inflammation. The TLR-2 recognizes microbial components like lipoprotein, lipoteichoic acid, and peptidoglycan. An association of *TLR2*-196 to -174 del polymorphism with susceptibility to gastric cancer was demonstrated in Brazilian population (Table 3; de Oliveira and Silva, 2012). The genotypes *TLR2* ins/del and del/del were found more often in the individuals with gastric cancer than the healthy individuals (de Oliveira and Silva, 2012). This polymorphism is also associated with increased risk of non-cardia gastric cancer in Japan (Tahara et al., 2007b). A 22-bp deletion in *TLR2* gene that alters the promoter activity decreases transcriptional activity of the gene. Similarly, the Asp299Gly (rs4986790) and Thr399Ile (rs4986791) polymorphisms of *TLR4* gene were shown to be the risk factors of gastric carcinoma in Caucasian and Indian populations (Table 3; Jing et al., 2012), although these polymorphisms are rare in Japanese and Chinese populations (Tahara et al., 2007a). Rather, the Chinese individuals with *TLR5* rs5744174C carriers (TC+CC), but not with the TT genotype, have the increased risk of gastric cancer (Zeng et al., 2011). Likewise, the SNPs associated with TLRs (e.g., *TLR1* rs4833095 and *TLR10* rs10004195) also contribute to the susceptibility to *H. pylori* infection and may influence the clinical outcomes (Table 3; Simawaranon et al., 2017).

**Table 3:** Human genes mediating immune response and their polymorphisms associated with risk of gastric diseases.

Genes	Function	Polymorphisms associated with gastric diseases	Reference
*TLR1*	Induce cytokine secretion	CC and TT genotype of TLR1 rs4833095	Simawaranon et al., 2017
*TLR2*	Recognizes acylated bacterial lipoproteins and signals	-196 to −174 del	de Oliveira and Silva, 2012
*TLR4*	Acting as a receptor for lipopolysaccharide (LPS),elicits immune response	Asp299Gly (rs4986790), Thr399Ile (rs4986791)	Tahara et al., 2007a
*TLR5*	Recognize bacterial flagellin.	rs5744174C carriers (TC+CC)	Zeng et al., 2011
*TLR10*	Suppress inflammatory signaling on primary human cells	AA genotype of TLR10 rs10004195	Simawaranon et al., 2017
*NOD-1*	Intracellular recognition molecules for pathogen-associated molecules	rs7789045 TT and rs2709800 TT genotypes	Wang et al., 2012
*NOD-2*	Intracellular recognition molecules for pathogen-associated molecules	R702W (SNP8)	Rosenstiel et al., 2006
*IL-1β*	Key proinflammatory cytokine in gastric mucosa	-511C/T	Li et al., 2007a
*IL-1RN*	Inhibits the activity of IL-1	*IL-1RN*2/2	El-Omar et al., 2000
*IL-2*	Promotes differentiation of T cells	−330T>G (rs2069762), +114TT	Melchiades et al., 2017
*IL-4*	Induces differentiation of naive helper T cells to Th2 cells.	−590T, −33TT	Sampaio et al., 2015
*IL-6*	Regulates the immune system.	−174C>G	Sampaio et al., 2015
*IL-8*	Promoter of angiogenesis, act as a chemoattractant	−251T>A	Wang et al., 2018
*IL-10*	Anti-inflammatory cytokine.	GCC haplotype	Wu et al., 2003
*IL-18*	Inflammatory cytokine facilitates type 1 immunity	rs187238 (−137G>C), rs360718 (113T>G), rs360717 (127C>T)	Myung et al., 2015
*TNF-α*	Key immune mediator against Gram negative bacteria	GA, AA and GA+AA genotypes of TNF-α-308	Xu et al., 2017

The activation of another intracellular PRR, the nucleotide-binding oligomerization domain 1 (NOD-1), leads to the production of pro-inflammatory cytokines. Several studies suggested that the polymorphisms in *nod-1* gene, the rs7789045 TT and rs2709800 TT genotypes, are linked to increased risk of gastric cancer (Table 3; Wang et al., 2012). The NOD-1 deficient mice are more prone to get infected with *cagA* positive strains, and the NOD-1 mutants produce lower amount of macrophage inflammatory protein-2 (MIP-2 or CXCL-2) than the wild type. The individuals carrying the R702W(SNP8) mutation in *NOD-2/CARD15* gene failed to recognize the *cag*PAI-positive *H. pylori* (Table 3; Rosenstiel et al., 2006).

The mutations in the promoter and the transcribed regions of pro-inflammatory and anti-inflammatory cytokine genes lead to impaired expression of cytokines and aberrant responses to the invading pathogens. In the context of gastric physiology, IL-1β is a potent acid inhibitor and modulates various biological functions of gastric epithelial cell types. Upon *H. pylori* infection, IL-1β induces the pro-inflammatory immune response along with the inhibition of gastric acid leading to hypochlorhydria, which facilitates bacterial colonization. The functionally relevant polymorphisms in *IL-1β* promoter region could be associated with high or low IL-1β production. The *IL-1β*-511T+, *IL-1β*-31C+, and *IL-1RN* 2/2 polymorphisms are shown to be associated with the increased risk of hypochlorhydria and gastric cancer (El-Omar et al., 2000). In Chinese population, the individuals carrying the *IL-1β*-511C/T genotypes (CT carriers) have an increased risk of developing gastric cancer (Li et al., 2007a). For the Japanese population, it was shown that the carriers of the *IL-1β-*511T/T genotype or *IL-1RN*2/2 alleles had higher mucosal IL-1β than non-carriers (Table 3; Hwang et al., 2002). The presence of a variant GG genotype of *IL-2* gene at −330T>G (rs2069762), and +114TT SNPs were found to be associated with an increased risk of gastric cancer in *H. pylori* infected Brazilian population (Melchiades et al., 2017). Similarly, a higher prevalence of *IL-4* −590T and *IL-4* −33TT genotypes was identified among the intestinal-type gastric cancer patients. For the Romanian population, the *IL-4R −3223CÆT* polymorphism has been shown to be associated with gastric cancer (Burada et al., 2012). Those, who carry the *IL-4R −3223TT* genotype, have 2.5-fold higher risk of developing gastric cancer (Table 3; Burada et al., 2012). The higher production of *IL-6*, a multifunctional cytokine, found to be associated with the −174C>G allele as compared to the CC genotype. The prevalence of *IL-6* −174CG genotype is higher among the Portuguese patients with intestinal-type gastric cancer (Table 3; Sampaio et al., 2015). Likewise, the*IL-18* SNPs, rs187238 (−137G>C), rs360718 (113T>G), and rs360717 (127C>T) genotypes were linked to the *H. pylori-* associated diseases (Myung et al., 2015). IL-8 is considered as a potential determinant in inflammation, tumor progression, metastasis, and angiogenesis (Lee et al., 2013). The presence of T-251A polymorphism in the *IL-8* gene was shown to be associated with *H. pylori* infection and peptic ulcer (Table 3). Several studies showed that the subjects who are genetically predisposed to the enhanced production of anti-inflammatory cytokine IL-10 (particularly those who carry the GCC haplotype) are at higher risk of developing gastric cancer (Table 3; Wu et al., 2003; Sugimoto et al., 2007; Wang et al., 2018). TNF-α is a potent proinflammatory cytokine which is involved in the pathogenesis of *H. pylori*-associated gastric diseases (Thalmaier et al., 2002). Among the various polymorphisms in the TNF-α gene, GA, AA, and GA + AA genotypes of TNF-α-308 were identified to be significantly associated with gastric cancer when compared to homozygous GG type in Chinese population (Table 3; Xu et al., 2017).

Apart from cytokines, the dysregulation in the cellular and molecular pathways like survivin, COX-2, CDH1, and p53 also add on to higher risk of gastric cancer (Valenzuela et al., 2010). The G>A (−899) polymorphism in the *cox-2* gene is related to the hyper-inflammatory responses. Similar to *cox-2*, mutations in the tumor suppressor gene, epithelial cadherin (E-cadherin) is linked to the hereditary diffuse gastric cancer (Liu and Chu, 2014). Mutations in the gene encoding E-cadherin leads to increased cell proliferation, migration, and reduced apoptosis Studies revealed that the promoter hyper-methylation of the E-cadherin gene has a significant role in gastric cancer (Miyazaki et al., 2007; Liu and Chu, 2014). The p53 is a tumor suppressor protein encoded by the *TP53* gene, which is one of the most mutated genes related to cancers. The homozygous Pro/Pro genotype of the p53 codon 72 polymorphism increases the risk for gastric cancer (Yi and Lee, 2006). The results of a case control study in Chilean population showed that the polymorphisms in the proteins of the RAS/RAF/MEK/ERK pathways, such as rs3729931 (RAF1), rs45604736 (HRAS), rs2283792, and rs9610417 (MAPK1), are associated with gastric cancer (Gonzalez-Hormazabal et al., 2019).

## Gastrointestinal Microbiota

In the GI tract, stomach has the lowest and colon has the highest number of microbes. Most of these microbes are not easy to grow in laboratory due to the lack of appropriate conditions. However, our initial knowledge on the microbial population in stomach was derived from the culture based methods, which showed that apart from *H. pylori*, human stomach is the niche of many other bacteria that belong to the Firmicutes, Proteobacteria, Actinobacteria, and Fusobacteria phyla (Savage, 1977; Adamsson et al., 1999). Then a culture-independent, cloning and sequence based early metagenomic study showed that the gastric microbiota are mainly dominated by the members of five bacterial phyla: Proteobacteria, Firmicutes, Bacteroidetes, Actinobacteria, and Fusobacteria (Bik et al., 2006). This was followed by a number of studies based on newer Phylochip hybridization array analysis and 454-pyrosequencing, which showed thathuman stomach, when it is not colonized with *H. pylori*, harbors a diverse array of microbes that belong mainly to phyla like Streptococcus, Proteobacteria, Bacteroidetes, Actinomyces, Prevotella, and Gemella (Andersson et al., 2008; Maldonado-Contreras et al., 2011). But, the human stomach that carries the burden of *H. pylori* infection, also suffer from dysbiosis, which is manifested by the decrease of microbial diversity, lower relative abundance of Actinobacteria and Firmicutes, and higher relative abundance of Proteobacteria other than *H. pylori* (Phukan et al., 2006; Handa et al., 2010; Das et al., 2017). This seems reasonable since the long-term colonization of *H. pylori* initiates the inflammatory cascades leading to atrophic gastritis, loss of the acid secreting parietal cells, and eventually an elevation of the gastric pH, which may preferentially allow certain microbes to proliferate faster in the altered gastric milieu and establish colonization (Figure 1). Some of these invading species, particularly the pathogenic ones that belong to the Proteobacteria phylum, may also facilitate the development of gastric diseases including cancer through various mechanisms like enhancement of inflammation, stimulation of cell proliferation, modification of stem cell dynamics, and production of toxic metabolites (Lofgren et al., 2011; Abreu and Peek, 2014). Recent studies using modern sophisticated next-generation sequencing techniques like Illumina have helped to understand the association between the composition of gut microbiota and the pathogenesis of various disease conditions. For example, a recent study revealed that patient groups having either gastric carcinoma or chronic gastritis have distinct microbiota compositions (Ferreira et al., 2018). In contrast to the patients with chronic gastritis, the patients with gastric cancer have lower abundance of *Helicobacter* and *Neisseria*, while the abundance of *Achromobacter*, *Citrobacter*, *Phyllobacterium*, *Clostridium*, *Rhodococcus*, and *Lactobacillus* was higher; Ferreira et al., 2018). Another study involving the populations of Singapore and Malaysia showed that several bacterial taxa, including *Veillonella*, *Lactococcus*, and *Fusobacteriaceae* were enriched in the stomach of patients with gastric cancer (Castaño-Rodríguez et al., 2017). The microbial compositions and the bacterial interactions possibly differ in different stages of gastric carcinogenesis (Coker et al., 2018). The *Streptococcus*, *Prevotella*, and *Neisseria* were found highly abundant in chronic gastritis patients along with *H. pylori*. However, for the patients with gastric carcinoma, the abundance of *Helicobacter* were less, while several taxa like *Citrobacter*, *Clostridium*, *Lactobacillus*, *Achromobacter*, and Rhodococcus were found to be more abundant (Ferreira et al., 2018). Most of them are commensals but also have the potentials to become opportunistic pathogens (Ferreira et al., 2018). A study from Taiwan also showed evidence of *Clostridium* and *Fusobacterium* colonization in the stomach of patients with gastric cancer (Hsieh et al., 2018).

Like gastric microbiota, the significance of intestinal microbiota in the context of *H. pylori* infection and its related gastric diseases have also been tested and verified recently. In murine model, it has been shown that *H. pylori* colonization in stomach is capable of altering the intestinal microbiota (Kienesberger et al., 2016). In human, for the Chinese and Indian populations, *H. pylori* infection in stomach leads to an increase in the microbial diversity in the intestine (Figure 1; Gao et al., 2018; Devi et al., 2021). This increased diversity in the intestinal microbiota for the *H. pylori* infected individuals could be due to the increased pH of the stomach or could be due to the altered gut immunity. In the Chinese population, a decrease in the abundance of Bacteroidetes and an increase in the abundance of Firmicutes and Proteobacteria were observed in the intestine of the *H. pylori* infected individuals (Gao et al., 2018). In Indian population, for the *H. pylori* infected individuals, the relative abundance of genus *Prevotella* and genus Dialister were found to be higher, while the relative abundance of the genus *Bifidobacterium* and genus *Bacteroidetes* were found to be lower in the intestine (Devi et al., 2021). Most remarkably, the *H. pylori* infected patients who had developed either gastric ulcer or gastric cancer, had a significantly low relative abundance of several species of *Bifidobacterium* (e.g., *B. Longum* and *B. adolescentis*) in the intestine (Table 4; Figure 1; Devi et al., 2021). It is worth mentioning that the bacteria belong to genus *Bifidobacterium* are mostly intestinal commensal bacterium and some of them carry anti-*H. pylori*, anti-cancer, and anti-ulcer activities. Also, several of the *Bifidobacterium* strains are known probiotics and provide benefit to the host by reducing inflammation.

**Table 4:** Beneficial microbes present in human gastric microbiota and intestinal microbiota.

Bacterium	Effects	Reference
*Bifidobacterium*	Anti-*H. pylori*, anti-cancer, anti-inflammatory and anti-ulcer activities	Devi et al., 2021
*Lactobacillus casei*	Inhibits the urease activity and reduce colonization of *H. pylori* in the stomach	Sgouras et al., 2004
*Lactobacillus rhamnosus*, GG strain and yoba 2012 strain	Alleviates *H. pylori* induced apoptosis and inflammation	Westerik et al., 2018
*Lactobacillus acidophilus*	Reduces *H. pylori* induced inflammation	Bhatia et al., 1989; Yang et al., 2012

A number of commensal microbes in the gut provide essential health benefits to its host in different ways (Table 4). Many of them have protective effect against *H. pylori* and therefore their abundance is crucial. Some of the commensals have also been used as probiotics, which utilize mechanisms like production of antimicrobials and antioxidants that can inhibit urease, compete with *H. pylori* to bind the surface of gastric epithelial cells, block their specific membrane receptors, and stabilize the mucosal barrier of the stomach by stimulating mucus production by surface epithelial cells (Khoder et al., 2016). Recent meta-analysis studies suggest that the probiotic supplementation along with antibiotic treatment for *H. pylori* eradication reduces the adverse side effects and improves the rate of eradication. Certain species of *Lactobacilli* are capable of preventing antibiotic-associated diarrhea along with the inhibition of potentially pathogenic species. *Lactobacillus acidophilus* has anti-*H. pylori* activity (Bhatia et al., 1989). Moreover, *L. acidophilus* was effective in suppressing the Smad7 and NF-kB pathways, and ameliorates the *H. pylori* induced inflammation (Yang et al., 2012). Likewise, probiotics such as *L. bulgaricus* and *L. salivarius* have the ability to reduce inflammation by the downregulation of IL-8 secretion (Qureshi et al., 2019). In addition, another *Lactobacillus* species, *L. casei*, inhibits the urease activity and reduces the colonization of *H. pylori* in stomach Table 4 (Sgouras et al., 2004). Furthermore, *L. reuteri* and *L. johnsonii* La1 inhibits the adhesion of *H. pylori* to the epithelium by competing for the specific binding site and secreting antimicrobial substances, respectively. Also, the reuterin produced by *L. reuteri* ATCC 55730 exhibits an inhibitory effect on the *vacA* gene expression of *H. pylori* (Qureshi et al., 2019).

Apart from this, a significant crosstalk exists between the gut microbiota and the host immune system. The short-chain fatty acids (SCFAs), produced as a result of microbial fermentation in gut, can activate extracellular signal-regulated kinase 1/2 and p38 mitogen-activated protein kinase signaling pathways in the epithelial cells resulting in production of cytokines and chemokines (Kim et al., 2013). The reduction in the SCFAs producing bacteria results in the T cell imbalance and dysregulations in the mucosal Th17 cells (Luu and Visekruna, 2019). The composition of GI microbiota is dynamic and the impacts of several other factors on the *H. pylori* associated gastric diseases could actually be through the alteration of GI microbiota.

## Diet

Diet is increasingly being recognized as a critical factor in determining the susceptibility of an individual toward alteration of GI microbiota, *H. pylori* infection and gastric diseases. For example, a case–control study in Ardebil city, Iran for 128 adults (42 gastric cancer and 86 healthy) showed that both *H. pylori* infection and diet had a significant relationship with gastric cancer (Table 5; Nemati et al., 2012). Consumption of salted food, red meat, processed or smoked meat, and fishes increase the risk of developing atrophic gastritis with intestinal metaplasia and gastric cancer. The *H. pylori*-infected stomach has a lower level of vitamin C (Zhang et al., 1998), reduced synthesis of mucin (Byrd et al., 2000), and an increased level of ROS (Handa et al., 2010). These effects could get aggravated with the higher consumption of red meat since it contains heme bound iron, which helps formation of hydroxyl radicals (OH•; Macho-González et al., 2020). The preservatives such as nitrites presents in the preserved meat increase the exogenous exposure to nitrosamines and N-nitroso compounds (NOC), which increase the risk of gastric cancer (Jakszyn and González, 2006). Therefore, high consumptions of red meat and processed meat like bacon, ham, and sausages create oxidative stresses by forming ROS, which may lead to chronic diseases like gastric cancer (Zhu et al., 2013). A study from the state of Mizoram in Northeast India showed that apart from *H. pylori* infection, consumption of smoked, dried, and salted meat and fish as well as fermented pork fat increase the risk of gastric cancer (Phukan et al., 2006). Excessive consumption of salt can also damage the lining of the stomach, enhance NOC formation, and can facilitate the *H. pylori* colonization (Fox et al., 1999). The High temperature requirement A (HtrA) protein of *H. pylori* enables the bacteria to survive under stresses, like extreme salt concentration, pH, and temperature (Hansen and Hilgenfeld, 2013). This is more clarified with a follow-up study on salt consumption and gastric cancer development involving 2,476 subjects for 14 years showing significant salt-gastric cancer associations (Table 5; Shikata et al., 2006). The high salt diet also enhanced the gastric damages by the CagA expressing *H. pylori* strains leading to carcinogenesis in Mongolian gerbils (Gaddy et al., 2013). Significant association was found between the gastritis and the consumption of beverages like coffee and tea although their association with gastric cancer remained inconclusive (Sanikini et al., 2015; Mahmoud et al., 2016). A study conducted in Korea demonstrated that the individuals who carry the (TCA+TCG) haplotype in *zip11* gene and consume spicy foods have 2.6-fold higher chance for developing chronic gastritis than the individuals with CAA haplotype in *zip11* gene (Ha and Bae, 2018).

**Table 5:** Impact of dietary elements on gastric health.

Dietary elements contributing to gastric diseases	References	Dietary elements with beneficiary effects on gastric health	References
Red meat and processed meat		Fruits	
*Heme in meat catalyze the superoxide and hydrogen peroxide conversion to hydroxyl radicals*	Macho-González et al., 2020	*Contains ascorbic acid, which scavenge reactive radicals and prevents DNA damage*	Drake et al., 1996
Nitrites in preserved meat (ham, bacon)		Dairy products	
*Nitrosamines, N-nitroso compounds (NOC), reactive oxygen species (ROS) formation*	Jakszyn and González, 2006	*Prebiotic property*	Kok and Hutkins, 2018; Rezac et al., 2018; Tamang et al., 2020
High salt consumption		Turmeric (curcumin)	
*Damages stomach lining, facilitates H. pylori colonization*	Fox et al., 1999	*Anti-inflammatory and anti-cancer properties*	Sarkar et al., 2016

On the other hand, several components in diet may have protective roles. The intake of fruits and vegetables reduce the risk of gastric cancer as well as other gastrointestinal disease (Wang et al., 2014). The ascorbic acid present in fruits helps to scavenge the reactive radicals generated in human gastric mucosa and thereby prevents the DNA damage (Drake et al., 1996). Many oligosaccharides like inulin-type fructans polysaccharides including dietary fibers benefit the GI tract with their prebiotic activities (Mitmesser and Combs, 2017). The Wheat Peptides and Fucoidan (WPF) and SCFA help to mitigate the progression of chronic superficial gastritis (Kan et al., 2020). The fermented foods like dairy products serve as a natural source for beneficial microbes and some of them also have anti-*H. pylori* effects (Table 5; Nair et al., 2016). Consumption of fermented foods like yogurt, buttermilk, kefir, and kimchi helps to restore the count of beneficial microbes (e.g., *Bifidobacterium*) in gut (Kok and Hutkins, 2018; Rezac et al., 2018; Tamang et al., 2020).

The data from several systematic reviews and meta-analysis of cohort studies show that the polyphenols present in fruits and vegetables have protective effect against type 2 diabetes and cardiovascular diseases (Del Bo et al., 2019). Further, the diet rich in polyphenols possess anti-inflammatory and antimicrobial properties and reduce oxidative stress by modulating immune pathways. Green tea is rich in polyphenol, catechin which is a potent antioxidant. Apart from this, fruits and vegetables rich in polyphenols can modulate gut microbiota by promoting the growth of beneficial gut microbes and inhibits pathogenic species (Cory et al., 2018).

The eating patterns of warm vs. cold countries are significantly different and that may also contribute to the variation in the susceptibility to gastric diseases. Bacteria grow more rapidly in the warmer climates and causes food spoilage. However, people living in warm climates generally uses more spices during cooking and many of these spices have antibacterial properties (Gutierrez and Simon, 2016). For example, turmeric, clove, oregano, thyme, cinnamon, and cumin are known to have antibacterial (against *Bacillus subtilis*, *Pseudomonas fluorescens*, *Staphylococcus aureus*, and *Vibrio parahaemolyticus*) and antifungal (against *Aspergillus flavus*) properties (Liu et al., 2017). The active ingredient of turmeric, curcumin, has anti-*H. pylori* activity in addition to its anti-inflammatory, neuroprotective, and anti-cancer properties and is proposed to be used as therapeutic agent for *H. pylori* induced gastric diseases (De et al., 2009; Sarkar et al., 2016).

## Medication

Several epidemiological studies emphasized the association of drugs with the gastric diseases. The extensive use of anti-reflux drugs, antacids, antiplatelets, selective serotonin reuptake inhibitors (SSRIs), and Non-steroidal anti-inflammatory drugs (NSAIDs) have the potential to predispose an individual to gastric diseases. Aspirin, a commonly used NSAID, enhances the plasma pro-inflammatory cytokines such as TNF-α leading to leukocyte infiltration in gastric mucosa and induces gastric ulceration (Table 6; Musumba et al., 2009). The proton pump inhibitors, such as omeprazole, pantoprazole, and histamine H_2_ receptors antagonists like cimetidine and ranitidine are used as effective acid suppressors in the treatment of acid related disorders (Table 6). However, the reduction of gastric acid by the PPIs may facilitate the colonization of pathogenic bacteria in the gut (Macke et al., 2020). The prolonged use of these drugs increases the possibility of altering the gastric microbiota and leading to gastric diseases including gastric cancer (Rodriguez et al., 2006; Mărginean et al., 2018). A study on Flemish cohort showed that 10% of the inter-individual variations in gut microbiota compositions may occur due to prolonged use of antibiotics, laxatives, benzodiazepines, and antidepressants (Falony et al., 2016; Doestzada et al., 2018). On the other hand, metformin, which is used for the treatment of type II diabetes, could also preferentially promote the growth of the bacteria (e.g., *Roseburia* and *Subdoligranulum*) producing the SCFAs like butyrate (Table 6; Forslund et al., 2015; Doestzada et al., 2018). Like the medications perturb the GI microbiota, the microbiota can also affect the activity of a drug. For instance, some prodrugs such as sulfasalazine, which is used for treatment of ulcerative colitis, require bioactivation by the gut microbes (Kim, 2015).

**Table 6:** Drugs that affect gastric health and their mode of action.

Drug	Action	Association with gastric disease	Reference
Ranitidine, Cimetidine	Acid suppression	Increases the risk of gastric adenocarcinoma	Rodriguez et al., 2006
Omeprazole, Pantoprazole	Proton pump inhibition	Positive association with gastric diseases.	Mărginean et al., 2018
Metformin	Blood sugar control(Diabetes treatment)	Alleviate gastric disease risk by increase the abundance of bacteria that produce short-chain fatty acids (SCFAs)	Forslund et al., 2015
Aspirin	NSAIDS; enhance the secretion of pro-inflammatory cytokines	Positively associated with gastric ulcer	Musumba et al., 2009

Antibiotics like gramicidin, neomycin, bacitracin, and tyrothricin, which are used for sore throat has been found to develop antimicrobial resistance in different human pathogens (Essack et al., 2019). Emergence of the antibiotic resistant strains of *H. pylori* allows the bacterium to persist in the stomach for prolonged duration and eradication of the *H. pylori* infection becomes challenging. It is one of the 12 antibiotic-resistant “priority pathogens” and included under the “high priority” category by WHO (Tacconelli et al., 2018). Easy availability and indiscriminate use of antibiotics may result in enhanced antibiotic resistance in the present *H. pylori* strains. The global levels of *H. pylori* resistance (17.2% for clarithromycin, 26.7% for metronidazole, 11.2% for amoxicillin, 16.2% for levofloxacin, and 5.9% for tetracycline) are alarming (De Francesco et al., 2010). Thus, emergence of antibiotic resistance in *H. pylori* due to indiscriminate use of antibiotics and failure to eradicate the infection is a significant factor that contributes to the clinical outcomes.

## Discussion

*Helicobacter pylori* is a definite carcinogen with proven capabilities to trigger gastric adenocarcinoma and gastric MALT-lymphoma. It also has the potential to induce other severe and often terminal illnesses like duodenal and gastric ulcers. However, only a minor fraction of the people infected with the virulent *H. pylori* strains develops severe gastric diseases, while most of the infections remain benign indicating involvement of multiple factors. The most studied factor in this context is the variations in the virulence factors of *H. pylori*. For instance, the binding affinities of *H. pylori* may differ among strains because of the variations in the BabA sequences affecting the adhesion to the gastric epithelium. Similarly, certain polymorphisms in the *vacA* and the *cagA* genes may result in highly virulent combinations of the expressed proteins. Infections with the strains carrying such allelic combinations (e.g., *vacAs1m1cagA*+) have a higher risk of developing aggressive diseases than the infections with strains carrying the less virulent allelic combinations (e.g., *vacAs2m2cagA*-). Moreover, *H. pylori* infection, apart from causing gastric epithelial damage, also alters the gastric niche leading to loss of active *H. pylori* infection and it was suggested that *H. pylori* causes gastric cancer in the “hit and run” mechanism (Hatakeyama, 2014). However, even though its contribution to cause gastric illness is well-established, in many cases, *H. pylori* infection alone is insufficient to induce severe gastric diseases. Here, we showed that the contributions from other etiological factors in determining the clinical outcomes cannot be denied (Figure 1).

The polymorphisms in host genes encoding the immune effector proteins play important roles in the inter-individual variations in clinical outcomes since they directly add on to the susceptibility of an individual to *H. pylori* infection and related diseases. It is also evident that diet has a major role in either protecting an individual from gastric diseases or predisposing the individual to gastric pathologies. The enhanced oxidative stresses due to the consumptions of red meats may lead to chronic gastritis. Moreover, the NOC, which is formed by the consumption of salted food, damages the lining of stomach and facilitates the *H. pylori* induced gastric damages. This, on the other hand, can be mitigated by the protective effect of the WPF from wheat and the SCFA produced by the probiotics and even by the commensal microorganisms that are commonly present in the ethnic fermented foods (Nair et al., 2016). The intake of prebiotics may also be beneficial in preventing the development of severe gastric diseases. Conversely, the prolonged acid suppressions caused by the drugs like ranitidine, cimetidine, and omeprazole may increase the risk of gastric adenocarcinoma (Rodriguez et al., 2006). The extensive use of modern medicines may also lead to dysbiosis in the GI microbiota, which is a major factor in determining the susceptibility of several diseases including peptic ulcer and gastric cancer (Blaser, 2014).

*Helicobacter pylori* colonization in stomach also leads to dysbiosis of gastric and intestinal microbiota. Increased abundance of certain bacteria (e.g., bacteria belonging to the phyla Proteobacteria and Fusobacteria) in the stomach upon dysbiosis may have the potential to contribute in the process of ulceration and carcinogenesis. In direct contrast, several bacterial genera in the stomach (e.g., *Lactobacillus*) and in the intestine (e.g., *Bifidobacterium*) have the ability to protect the stomach from *H. pylori* infection and gastric diseases. Although poorly studied, but appears reasonable, it could be the ability of *H. pylori* to alter the GI microbiota that made the bacterium linked to a wide number of non-GI diseases (Table 1). For example, the recent studies showed the involvement of dysbiosis in the context of asthma and cardiac illness (Tang et al., 2017; Frati et al., 2019).

We conclude that even though *H. pylori* is the primary cause of the severe gastric diseases, the clinical outcomes are greatly influenced by “other factors” like host genetic polymorphisms, GI microbiota, diet, medication, geography, and climate. Their discrete as well as synergic effects predispose the *H. pylori* infected individuals to certain gastric diseases and also determine the severity of such diseases.

## Author Contributions

SC conceived the idea. SA, RR, DC, TD, and SD contributed in writing the manuscript. KD, NT, JT, SL, and SC have edited the manuscript. All authors contributed to the article and approved the submitted version.

## Conflict of Interest

The authors declare that the research was conducted in the absence of any commercial or financial relationships that could be construed as a potential conflict of interest.

## Publisher’s Note

All claims expressed in this article are solely those of the authors and do not necessarily represent those of their affiliated organizations, or those of the publisher, the editors and the reviewers. Any product that may be evaluated in this article, or claim that may be made by its manufacturer, is not guaranteed or endorsed by the publisher.
